# Development of a Novel Prognostic Nomogram for High Model for End-Stage Liver Disease Score Recipients Following Deceased Donor Liver Transplantation

**DOI:** 10.3389/fmed.2022.772048

**Published:** 2022-03-03

**Authors:** Mengfan Yang, Abdul Rehman Khan, Di Lu, Xuyong Wei, Wenzhi Shu, Chuanshen Xu, Binhua Pan, Zhisheng Zhou, Rui Wang, Qiang Wei, Beini Cen, Jinzhen Cai, Shusen Zheng, Xiao Xu

**Affiliations:** ^1^Department of Hepatobiliary and Pancreatic Surgery, Affiliated Hangzhou First People's Hospital, Zhejiang University School of Medicine, Hangzhou, China; ^2^National Health Commission Key Laboratory of Combined Multi-Organ Transplantation, Hangzhou, China; ^3^Organ Transplantation Center, The Affiliated Hospital of Qingdao University, Qingdao, China; ^4^National Center for Healthcare Quality Management in Liver Transplant, Hangzhou, China; ^5^Department of Hepatobiliary and Pancreatic Surgery, Shulan (Hangzhou) Hospital, Hangzhou, China; ^6^Institute of Organ Transplantation, Zhejiang University, Hangzhou, China; ^7^Department of Hepatobiliary and Pancreatic Surgery, The First Affiliated Hospital, Zhejiang University School of Medicine, Hangzhou, China

**Keywords:** liver transplantation, model for end-stage liver disease score, cold ischemia time, ABO blood type incompatibility, graft survival

## Abstract

**Background:**

A high model of end-stage liver disease (MELD) score (>30) adversely affects outcomes even if patients receive prompt liver transplantation (LT). Therefore, balanced allocation of donor grafts is indispensable to avoid random combinations of donor and recipient risk factors, which often lead to graft or recipient loss. Predictive models aimed at avoiding donor risk factors in high-MELD score recipients are urgently required to obtain satisfactory outcomes.

**Method:**

Data of patients with MELD score >30 who underwent LT at three transplantation institutes between 2015 and 2018 were retrospectively reviewed. Early allograft dysfunction (EAD), length of intensive care unit (ICU) stay, and graft loss were recorded. Corresponding independent risk factors were analyzed using stepwise multivariable regression analysis. A prediction model of graft loss was developed, and discrimination and calibration were measured.

**Results:**

After applying the exclusion criteria, 778 patients were enrolled. The incidence of EAD was 34.8% (271/778). Donor graft macrovesicular steatosis, graft-to-recipient weight ratio (GRWR), warm ischemia time (WIT), cold ischemia time (CIT), and ABO blood incompatibility, together with donor serum albumins, were independent predictors of EAD. The incidence of ICU stay over 10 days was 64.7% (503/778). Donor age, recipient's MELD score, Child score, and CIT were independent predictors of ICU stay. The 3-year graft survival rates (GSRs) in the training and validation cohorts were 64.2 and 59.3%, respectively. The independent predictors of graft loss were recipient's Child score, ABO blood type incompatibility, donor serum total bilirubin over 17.1 μmol/L, and cold CIT. A nomogram based on these variables was internally and externally validated and showed good performance (area under the receiver operating characteristic curve = 70.8 and 66.0%, respectively). For a recipient with a high MELD score, the avoidance of ABO blood type incompatibility and CIT ≥6 h would achieve a 3-year GSR of up to 78.4%, whereas the presence of the aforementioned risk factors would decrease the GSR to 35.4%.

**Conclusion:**

The long-term prognosis of recipients with MELD scores >30 could be greatly improved by avoiding ABO blood type incompatibility and CIT ≥6 h.

## Introduction

Liver transplantation (LT) is an effective and curative treatment for patients with end-stage liver disease (ESLD). However, the pre-transplant state of these patients and the shortage of donor livers largely limit the development of LT. Many patients die due to long waiting lists or the unavailability of a suitable standard criteria donor (SCD) graft. Therefore, the allocation of organ resources has recently gained much attention. The model for end-stage liver disease (MELD) score of ESLD severity was adopted as a criterion for organ allocation (1, 2). As a comprehensive assessment tool for donor liver allocation, the MELD score is expected to address the drop-out risk of patients on the waiting list as well as predict post-LT mortality. The mortality rate during the waiting list period has decreased by a remarkable 30% after the introduction of the “sickest-first policy” based on MELD scores. Patients with high MELD scores have significantly higher morbidity and mortality, as well as longer intensive care unit (ICU) and hospital stays (3–5). For pediatric LT, the significant role played by the MELD score in predicting 3-year patient survival was also demonstrated (6).

The concept of extended criteria donor (ECD) has long been introduced to bridge the gap between the demand and supply of liver donor grafts. ECD is defined as donor age >60 years, hepatitis C virus (HCV)-positive donor, liver with cold ischemia time (CIT) >12 h, donor after cardiac death (DCD), or donor liver with macrovesicular steatosis (MVS) >30%. While liver grafts with MVS, DCD, or long CIT are being used for low-acuity recipients (7, 8) whether these ECD grafts could be accepted for high acuity patients with MELD scores over 30 remains to be determined. Previously, the use of ECD grafts has been reported to be associated with decreased survival among high-risk patients. As the MELD score does not consider the quality of the graft, it may not be an adequate tool for the utilization and allocation of ECD grafts. A number of other recipient risk factors related to outcome need to be explored, and the role of donor and surgical factors remains elusive.

The aim of this multicenter study was to evaluate donor risk factors for early allograft dysfunction (EAD), length of ICU stay, and graft loss in patients with high MELD scores. In addition, we aimed to identify the risk factors for complicated outcomes and higher costs. To strengthen the results of the analysis, a nomogram was established to predict graft loss. These findings may be used in the selection of liver graft donors and recipients and to predict graft survival (GS) in the high acuity cohort, thus improving post-transplantation prognosis.

## Materials and Methods

### Study Population

We reviewed medical records of patients who received deceased donor liver transplant (DDLT) at three transplantation centers in China, namely the First Affiliated Hospital of Zhejiang University School of Medicine, the Qingdao University Affiliated Hospital, and the Shulan (Hangzhou) Hospital, from January 2016 to July 2019. These records were collected and maintained by the China Liver Transplant Registry database.

Recipients with a MELD score >30 before LT were enrolled in this study. Cases of pediatric LT, split/reduced-size LT, re-transplantation, mortality during LT, abdominal multi-visceral transplantation, and combined liver and kidney transplantation were excluded.

To determine predictors of early allograft function, length of ICU stay, and GS, the following donor variables were analyzed: donor age, sex, body mass index (BMI), ABO blood type, CIT, warm ischemia time (WIT), and results of pre-procurement serologic tests (sodium [Na], potassium [K], blood urea nitrogen [Bun], creatinine [Cr], alanine aminotransferase [ALT], aspartate aminotransferase [AST), albumin [ALB), and total bilirubin [TB) levels).

LT was approved by the Liver Transplantation Committee of each institution and was performed after informed consent was obtained from all patients. This study was approved by the ethics committee and was performed according to the ethical principles of the Declaration of Helsinki. the requirement for informed consent related to the study was waived due to its retrospective nature. No organs from prisoners were used in the study.

### Definitions

EAD was defined by one or more of the following criteria: (I) TB ≥10 mg/dL on postoperative day (POD) 7; (II) international normalized ratio (INR) ≥1.6 on POD 7; or (III) ALT or AST levels >2,000 U/L within the first 7 PODs. Each case was classified as “EAD” or “non-EAD” according to the criteria above. Fatty infiltration of the liver was differentiated into macrovesicular and microvesicular steatosis. Macro-vesicular steatosis (MVS) was categorized qualitatively into four groups according to the pathological examination: no MVS, mild (<30%), moderate (30–60%), or severe steatosis (≥60%). Livers with severe MVS were not used for LT to avoid primary non-function and graft loss.

### Protocol for Perioperative Immunosuppression

Patients in this cohort underwent orthotopic LT or piggyback LT based on hemodynamic stability, without splenectomy. During the operation, intravenous methylprednisolone and basiliximab were administered as immunosuppressants in all patients. After LT, triple immunosuppression based on a regimen including tacrolimus, mycophenolate mofetil (MMF), and methylprednisolone was administered. All patients infected with hepatitis B virus (HBV) were treated with either entecavir or tenofovir combined with hepatitis B immunoglobulin to maintain a high concentration of HBsAb after the operation.

### Statistical Analyses

The distribution of variables was assessed using the Shapiro-Wilk tests. Parametric continuous variables are presented as mean±standard deviation (SD), non-parametric distribution data as median and interquartile ranges (Q1–Q3) and discrete variables as numbers and percentages. The baseline characteristics were compared using *t*-tests and chi-square tests. Kaplan-Meier survival analysis was used to evaluate GS rates (GSR), and differences among survival curves were tested by log-rank tests. Two multivariable logistic regression models were applied to identify predictive factors for EAD and length of ICU stay over 10 days, using the predictive factors with *P* < 0.1 in the univariable analysis. The enrolled patients were divided into training and validation cohorts at a ratio of 1:1. Similarly, a multivariable Cox regression model was used to identify predictive factors for GSR. Based on the multivariable analysis, the nomogram for GS was formulated using the rms package in R. The discrimination of the nomogram was evaluated using the concordance index (C-index). Bootstraps with 1,000 resamples were used to validate the nomogram and construct a calibration curve. The Hosmer-Lemeshow (HL) goodness-of-fit test was used to assess the calibration of the model. The software program SAS (version 9.1) and R software (version 3.6.1) were used for statistical analysis, and statistical significance was set at *P* < 0.05.

## Results

### Patient Characteristics

During the study period, 2,210 patients with hepatocellular carcinoma (HCC) and liver cirrhosis at various stages were enrolled in the three institutions. A total of 939 patients had a MELD score >30, and 1,271 patients with MELD scores ≤ 30 were excluded. Furthermore, 46 cases of pediatric LT, 15 of split/reduced-size LT, 46 of re-transplantation, 6 of mortality during LT, 13 of combined liver and kidney transplantation, and 35 missing essential data were also excluded. After applying the inclusion and exclusion criteria, a total of 778 patients with MELD score >30 were collected and analyzed. The baseline characteristics of the patients are presented in Table 1. The median MELD score was 35 (interquartile range [IQR]: 32–40).

**Table 1:** Baseline characteristics of donor and recipients.

**Characteristics**	***n* = 778**
**Donor**	
Age (yr)	48.8 (40.6–56.9)
Gender, male	652 (83.8%)
**Steatosis**	
No	576 (74.0%)
Mild	173 (22.2%)
Moderate	29 (3.7%)
Donation type, DCD	304 (39.1%)
BMI (kg/m^2^)	22.8 (21.1–24.5)
Graft weight (g)	1,360 (1,205–1,560)
GRWR (%)	2.06 (1.77–2.44)
HB (g/dl)	11.1 (8.9–13.6)
WBC (×10^9^/L)	12.4 (9.4–16.3)
PLT (×10^9^/L)	147 (96–222)
Na (mmol/L)	145 (138–152)
K (mmol/L)	4.0 (3.7–4.5)
Bun (mmol/L)	7.4 (5.2–10.0)
Cr (μmol/L)	83 (59–128)
ALT (U/L)	38 (24–73)
AST (U/L)	56 (36–89)
ALB (g/L)	33 (29–37)
GGT (U/L)	50 (24–95)
ALP (U/L)	83 (64–111)
TB (μmol/L)	15.3 (10.5–21.6)
**Recipient**	
Age (yr)	50 (42–57)
Gender, male	653 (83.9%)
BMI (kg/m^2^)	23.0 (21.2–25.3)
**Liver disease etiology**	
Hepatitis B	599 (77.0%)
Hepatitis C	31 (4.0%)
Alcoholic	94 (12.1%)
Autoimmune	36 (4.6%)
Other	56 (7.2%)
MELD score	35 (32–40)
CP score	11 (10–12)
Waiting time (d)	34 (26–57)
Follow-up time (d)	534 (238–753)
**Perioperative**	
Operative time (h)	5.2 (4.5–6.5)
Anhepatic phase (min)	60 (46–77)
WIT (min)	4 (2–8)
CIT (h)	8.2 (7.2–10.7)
ABO blood type in compatible	151 (19.4%)
Intraoperative blood loss (ml)	1,000 (800–1,800)
Intraoperative blood transfusion (U)	4 (2–8)

### Perioperative Outcomes

The incidence of EAD was 34.8% (271/778) in the entire cohort. According to the univariable logistic regression analysis, donor graft MVS, graft-to-recipient weight ratio (GRWR), recipient sex, WIT, CIT, serum K > 5.5 mmol/L, ALB <28 g/L, and ABO blood incompatibility were identified as potential risk factors for EAD (*P* < 0.1, Table 2). All significant factors were included in the multivariable logistic model analysis. Donor graft MVS, GRWR, WIT, CIT, and ABO blood incompatibility, together with donor serum ALB <28 g/L, independently increased the odds of EAD.

**Table 2:** Univariate and multivariate logistic analysis of risk factors associated with early allograft dysfunction.

	***P*-value**	**HR**		**95%CI**	***P*-value**	**HR**		**95%CI**
Macrosteatosis	0.004				0.016			
Mild	0.099	1.538	0.922	2.565	0.442	1.253	0.705	2.227
Moderate	0.002	7.689	2.057	28.735	0.004	7.885	1.899	32.748
Recipient gender	0.093	1.620	0.922	2.846	0.369	1.371	0.688	2.732
GRWR (%)	0.000	2.282	1.484	3.509	0.008	1.984	1.192	3.304
WIT <5 (min)	0.000				0.002			
5 ≤ WIT <10 (min)	0.000	2.481	1.520	4.048	0.001	2.566	1.440	4.574
WIT≥10 (min)	0.000	2.745	1.560	4.830	0.009	2.485	1.253	4.928
CIT (h)	0.004	1.117	1.037	1.203	0.024	1.116	1.014	1.228
ABOi LT	0.008	2.055	1.208	3.496	0.020	2.106	1.124	3.945
ALB≥28 (g/L)	0.004	0.455	0.266	0.778	0.076	1.771	0.942	3.328

The incidence of ICU stay over 10 days was 64.7% (503/778) for this high MELD score cohort. Donor age, BMI, recipient age, MELD score, Child score, and CIT were identified as potential risk factors for ICU stay over 10 days according to the univariable logistic regression analysis (*P* < 0.1, Table 3). After inclusion in the multivariable logistic model analysis, donor age, recipient's MELD score, Child score, and CIT were shown to independently increase the odds of longer length of ICU stay.

**Table 3:** Univariate and multivariate logistic analysis of risk factors associated with length of intensive care unit stay over 10 days.

	***P*-value**	**HR**		**95%CI**	***P*-value**	**HR**		**95%CI**
MELD score	0.003	1.093	1.032	1.158	0.088	1.057	0.992	1.126
CP score	0.000	1.393	1.196	1.623	0.010	1.247	1.055	1.474
CIT (h)	0.006	1.116	1.032	1.208	0.000	4.491	2.227	9.056
Donor age≥60 (yr)	0.030				0.041			
50 < AGE ≤ 60 (yr)	0.350	1.309	0.744	2.303	0.393	1.306	0.707	2.414
40 < AGE ≤ 50 (yr)	0.024	2.031	1.097	3.762	0.035	2.062	1.054	4.034
AGE ≤ 40 (yr)	0.011	2.411	1.220	4.768	0.014	2.556	1.211	5.395

### Long-Term Graft Outcomes and Prediction Models for Graft Survival

**Figure 1:**
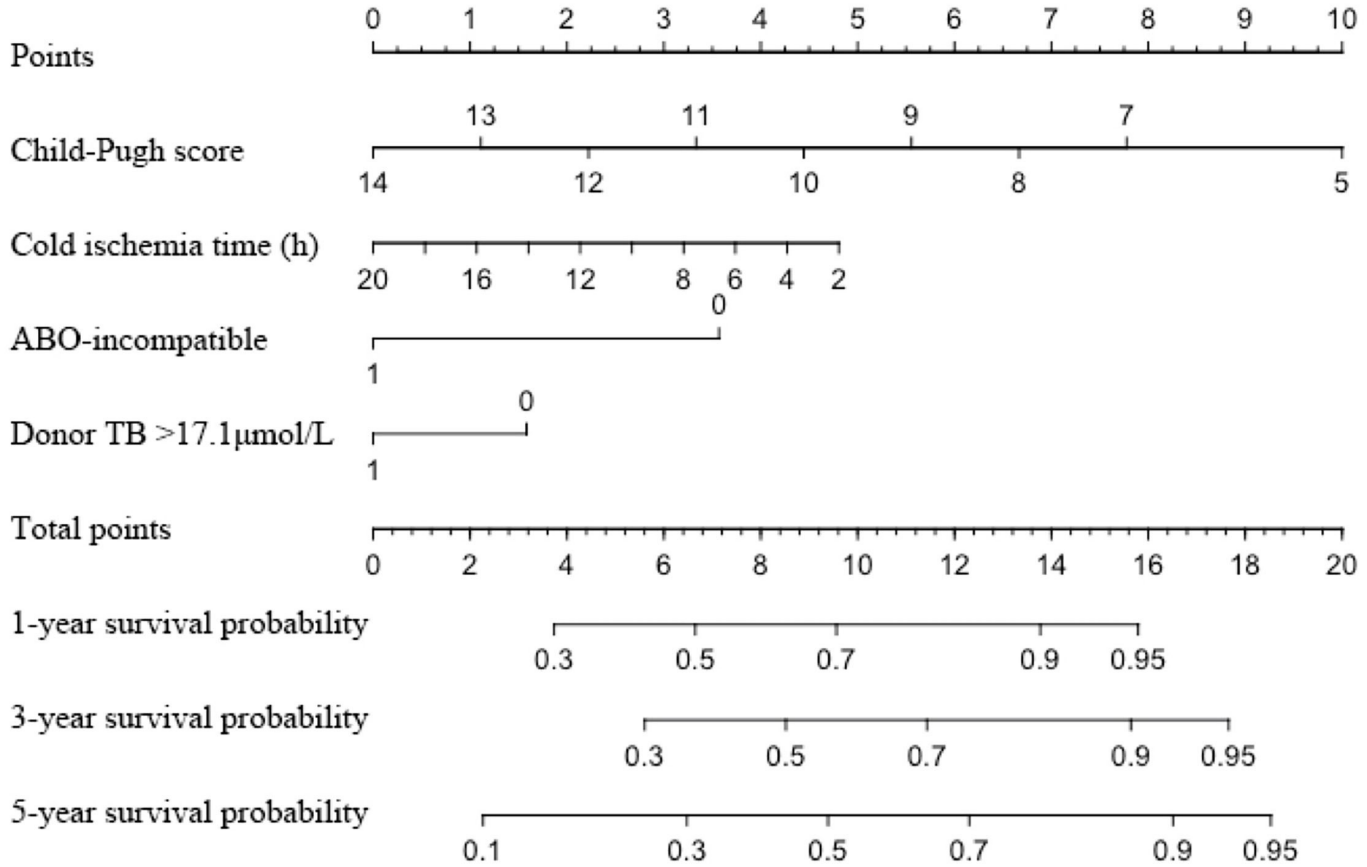
Nomogram for predicting the graft survival of recipients with MELD score over 30. MELD, model for end-stage liver disease; TB, total bilirubin.

**Figure 2:**
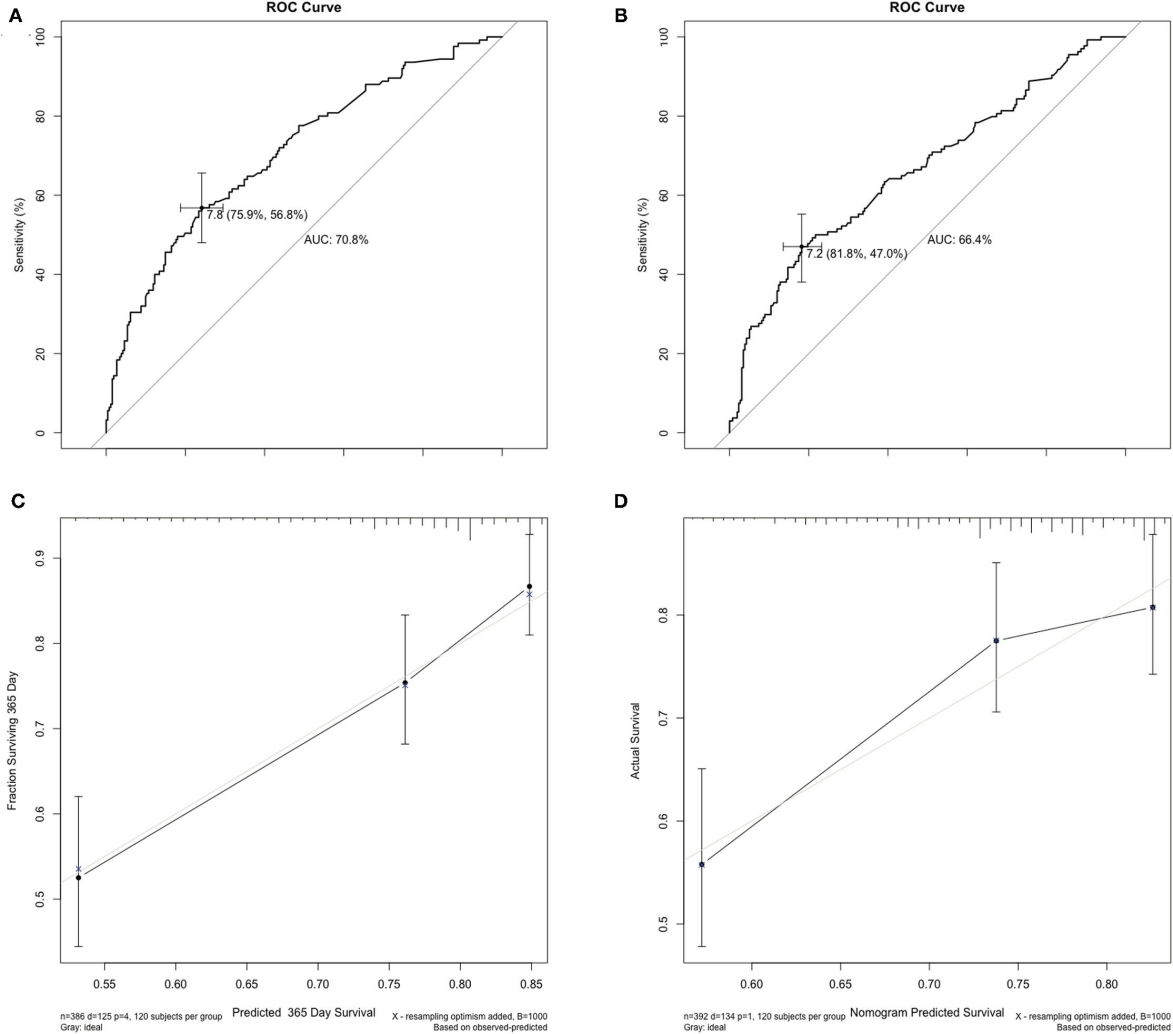
Internal and external validation of the AUC and the calibration curves for predicting the 1-year graft survival. **(A)** In the training cohort, the AUC for the established nomogram to predict graft survival was 0.708. **(B)** In the validation cohort, the AUC was 0.660. **(C,D)** The calibration curves for predicting incidence of 1-year graft survival rate following liver transplantation in the training and validation cohort. AUC, area under the receiver operating characteristic curve.

Two hundred and fifty-nine recipients encountered graft loss during follow-up (median follow-up 18 months, with 1-, 3-, and 5-year GSR of 71.5, 62.0, and 59.8%, respectively. The enrolled recipients were then assigned to the training and validation cohorts at a ratio of 1:1: the cohorts included 386 and 392 patients, respectively. The 3-year GSR in the training and validation cohorts was 64.2 and 59.3%, respectively. According to the univariable logistic regression analysis, recipient's Child score and BMI, WIT, CIT, donor serum TB >17.1 μmol/L and ABO blood incompatibility were identified as potential risk factors for graft loss (*P* < 0.1, Table 4). After being included in the multivariable Cox model analysis, recipient's Child score, ABO blood type incompatibility, and CIT were identified as independent predictors of graft loss (Figure 1). The nomogram based on these variables was established and internally and externally validated, showing good performance (area under the receiver operating characteristic curve [AUC] = 0.708 and 0.660, respectively; Figures 2A,B). The calibration plots demonstrated an outstanding agreement in the internal validation and an acceptable agreement between the nomogram prediction and the actual observation of GS in the external validation cohort (Figures 2C,D).

**Table 4:** Univariate and multivariate cox analysis of risk factors associated with graft loss.

	***P*-value**	**HR**		**95%CI**	***P*-value**	**HR**		**95%CI**
Recipient BMI (kg/m^2^)	0.075	0.952	0.901	1.005	0.150	0.963	0.915	1.014
CP score	0.000	1.380	1.203	1.584	0.001	1.260	1.102	1.440
WIT <5 (min)	0.094				0.184			
5 ≤ WIT <10 (min)	0.047	1.496	1.005	2.226	0.150	1.347	0.898	2.020
WIT≥10 (min)	0.110	1.461	0.918	2.325	0.665	0.895	0.541	1.480
CIT (h)	0.000	1.123	1.063	1.186	0.020	1.080	1.012	1.153
ABOi LT	0.000	2.985	2.056	4.334	0.000	2.449	1.660	3.612
TB≥17.1 (μmol/L)	0.013	1.562	1.100	2.219	0.071	1.392	0.972	1.992

### Subgroup Analysis for Graft Survival

**Figure 3:**
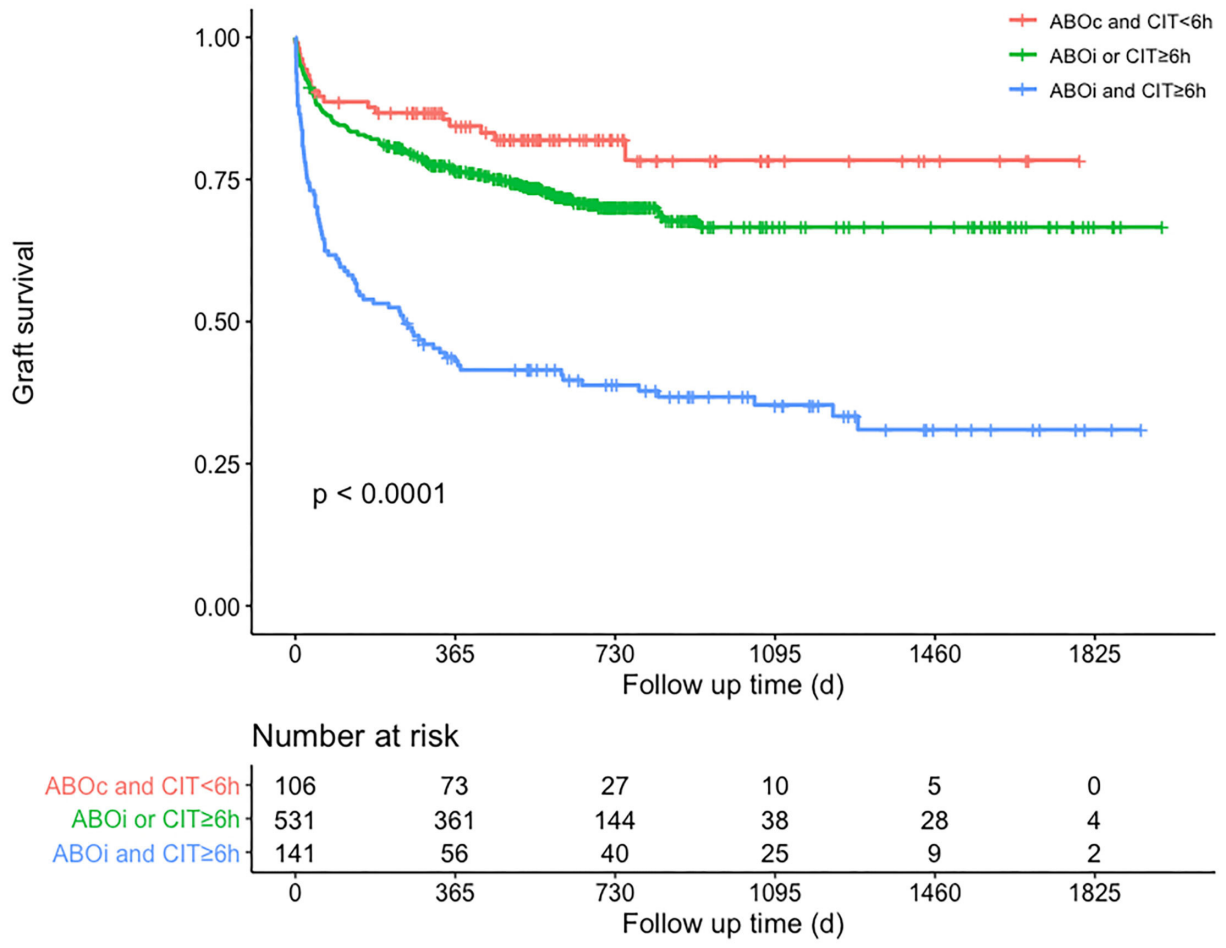
Patients' graft survival rates according to subgroup analysis of whether CIT is <6 h and ABO blood type is compatible. Comparisons between curves were performed with the log-rank test (*P* < 0.01). Red curve means that recipients received ABO compatible and CIT <6 h grafts, green curve means that recipients received ABO compatible and CIT ≥6 h grafts or received ABO incompatible and CIT <6 h grafts, blue curve means that recipients received ABO incompatible and CIT ≥6 h grafts. ABOc, ABO-compatible; ABOi, ABO-incompatible; CIT, cold ischemia time.

Subgroup analysis was performed on the basis of the Cox regression analysis. Recipients given an ABO blood type compatible liver graft with CIT <6 h (*n* = 106) would achieve a 3-year GSR of up to 78.4 %; however, those given an ABO blood type incompatible liver graft with CIT >6 h (*n* = 141) would have a lower 3-year GSR of 35.4% (Figure 3). Correspondingly, the incidence of EAD was 27.4% (29/106) and 51.1% (72/141), respectively, in these two groups (*P* < 0.01). Donor graft MVS did not affect the GSR, whether in mild or moderate degree, nor did the other donor serum parameters before liver procurement.

The 3-year GSR was 55.1 and 64.6%, respectively, in patients with or without EAD (*P* < 0.01). In addition, the 3-year GSR was 58.7 and 68.4%, respectively, for patients with or without longer length of ICU stay (*P* < 0.01).

## Discussion

The ever-growing need for liver grafts and the lack of available SCDs have pushed transplant centers to reconsider their selection criteria. This has led to the adoption of marginal or ECD grafts as a measure to endure, to some extent, the need-vs.-demand of donor liver grafts. Although ECD grafts can reduce the waiting time and its associated mortality, they carry associated risks such as EAD.

In this retrospective analysis, recipients with a relatively higher MELD score were enrolled, and independent predictors of graft loss were identified. These predictors were then combined to devise a nomogram for predicting graft loss. The subgroup analysis revealed that among patients with MELD score >30, ECD with incompatible ABO blood type, donor serum TB >17.1 μmol/L, and longer CIT liver grafts were independent predictors of graft loss. The presence of one or more of these factors will have an impact on GS. Our results are generally consistent with those of previous studies.

Although the MELD score allocation policy has decreased waiting list mortality, it has also reduced short-term survival rates, indicating that the MELD score is a strong outcome predictor (9). Schaubel et al. reported that high-risk grafts were beneficial for high MELD scores. Previously, there had been a trend of transplanting ECDs to low-MELD score patients, possibly based on the finding that patients with high MELD score were not in a better position to deal with the extra risks associated with ECD grafts (10). Immediately available ECD grafts achieve higher survival for patients with high MELD scores, as prolonged waiting list time and mortality risk in these patients may offset the higher risks of graft failure. Recently, it has been suggested that although high MELD score (≥35) patients with ECD graft LT have a higher rate of EAD, they still have no significant differences in terms of GS and rejection-free survival compared to SCD recipients (11).

The most common etiological cause of cirrhosis and HCC requiring LT in the Chinese population is HBV infection. These patients have frequent acute-on-chronic liver failure episodes and are more prone to sepsis and multi-organ dysfunction, leading to ultrahigh MELD scores before LT (12). To date, there is no unified model for evaluating prognosis after LT, especially for these high-acuity patients.

Since its inception and application, the MELD score has come a long way, and several refinements have been suggested to make it a better tool for predicting mortality in LT recipients. It has been proposed that refinements such as donor age × recipient MELD score (D-MELD) (13), or the difference between MELD score on listing and MELD score at transplant (Delta MELD), which takes into account dynamic alterations in disease severity during the waiting time, have better predictive power than the plain MELD score (14). Other prognostic scores exploiting the graft and recipient characteristics to better estimate post-transplant survival include the balance of risk score (BARS), the donor risk index (DRI), and the survival outcomes following LT (SOFT) (15, 16). These models do not show satisfactory accuracy and prediction efficiency. Still, patients with higher MELD scores at transplant were believed to be associated with a higher risk of mortality and graft failure (17, 18). Moreover, there is a direct association between the MELD score and the length of ICU/hospital stay post-LT (19).

Since high MELD score and ABO-incompatible (ABO-I) liver grafts are both independent risk factors for graft dysfunction and loss, combining these factors would negatively affect GS. It has also been observed that regardless of the combination, increasing the number of ECD graft factors would worsen the post-LT graft GS. This risk becomes more pronounced in high-risk patients, such as those with a high MELD score.

ABO-I LT without preoperative management leads to a cascade of cellular and antibody-mediated reactions that ultimately result in graft rejection. With the advent of B cell desensitization and reduction of preformed anti-donor ABO antibodies, ABO-I LT has become an important therapeutic option to increase the donor pool. ABO-I LT is usually limited to emergent situations when an ABO-compatible LT is unavailable, as it has been associated with an increased risk of graft loss. According to the OPTN/SRTR 2017 Annual Data Report, patients requiring urgent LT with MELD score >35 are already at increased risk of mortality while on the waiting list, and the use of an ABO-I ECD graft for LT compensates for the increased risk of low GS and EAD (20).

During LT, the liver graft is subjected to cold ischemia prior to implantation in the recipient. The cells most affected by ischemic injury are hepatocytes and liver sinusoidal cells, resulting in circulatory disorders and hepatocyte dysfunction, which manifest clinically as graft dysfunction. It has been reported that in patients with a high MELD score, the negative impact of long CIT was independent of the presence of other ECD factors such as age, and significantly decreased the 5-year post-LT survival (21). Moreover, CIT is associated with the post-LT length of hospital stay. (22) Lozanovski et al. (23) reported CIT >14 h, MVS >40%, and donor age >65 years to be independent risk factors for graft loss at 3-years when using ECD, and concluded that LT of high-risk grafts into high-risk individuals would yield poor outcomes.

Similarly, CIT has been reported to be associated with GS, length of post-transplant stay, and graft dysfunction. Recently, there has been a trend toward favoring shorter CIT because with longer CIT, the deleterious effects are more pronounced. Vladimir et al. observed that CIT should be kept within 10 h, otherwise it would lead to 1-year graft failure rates after LT of more than 25% (24). The tolerance for prolonged CIT decreased with an increasing MELD score. According to our multivariable Cox model analysis, the GSR of the recipients was the highest when the CIT was kept under 6 h, in agreement with results of previous studies (25). CIT, donor age, recipient's MELD score, Child score, and CIT were independent risk factors for longer ICU stay in the present study. Croome et al. also reported that while using ECD grafts, MELD score and CIT have an impact on the GS and that CIT should be kept <6 h and MELD score <30 (25).

While assessing a candidate for liver donation, the assessment of serum TB level is indispensable. Although many studies have concluded that recipients' high TB levels have a negative impact on post-LT outcomes, only a few studies have focused on the impact of donor TB levels. Briceno et al., in a proposal for scoring ECD liver grafts, suggested donor TB levels >2.0 mg/dL as one of the high risks for DDLT recipients (26). A number of studies involving donors with hyperbilirubinemia owing to Gilbert syndrome concluded that post-transplant hyperbilirubinemia could arise in recipients (27, 28). Another attempt at defining a liver graft index based on donor factors to establish the risk of graft failure stated that the risk of graft loss increases with donor age and TB level (29). Our results on the impact of donor TB levels are consistent with these findings as well. A donor serum TB exceeding 17.1 μmol/L is a risk factor for graft loss; however, the corresponding hazard ratio (HR) value indicated that its influence was not as intense as that of CIT or ABO-compatibility.

In view of the above findings, it is clear that random combinations of donor and recipient risk factors would increase the chances of EAD and graft loss in high MELD score recipients, and hence should be managed carefully when allocating ECD liver grafts to such patients. Since CIT is one of the factors that can be influenced, as opposed to donor age and MVS, it should be kept as short as possible to decrease the risk of GS and EAD. An outstanding problem is that the CIT cannot be estimated precisely before the completion of LT. Numerous studies have proposed novel techniques of machine perfusion to minimize the CIT and mitigate ischemic reperfusion injury (30, 31).

The recipient's Child score before transplant is another independent risk factor for graft loss, which can be managed with symptomatic and supportive treatment. Thus, patients on the waiting list should be assessed accordingly and treated appropriately to optimize their clinical status and improve their preoperative outcomes.

LT recipients often have a prolonged ICU stay. The length of ICU stay was directly proportional to the length of the overall hospital stay, and predicted the risk of GS and of future complications. We found that donor age, recipient's preoperative MELD score, child score, and CIT affected the postoperative length of ICU stay, which is partially consistent with the results of previous studies (32). However, since we selected patients with an ultra-high MELD score, and the median ICU stay time in the population was 10.3 days, we selected 10 days as the cut-off value to judge the ICU stay time in the present study. Previous studies have found that MELD score >24 and intractable ascites had an effect on the length of ICU stay. The length of the operation was also related to the length of stay in the ICU (32). Consistent with our conclusion, longer ICU stay is often associated with increased risk of graft loss and poor survival (33).

The primary limitation of this study is that, as in most multicenter retrospective analysis, large databases are subject to input errors and missing data. A large portion of the population was censored for the absence of preoperative serological indices. Another limitation is that all centers included in the study are located in the same region, so that donor and recipient characteristics probably do not reflect those of other countries, and some selection bias might be present. In addition, although sustaining acceptable outcomes while the definition of ECD is better quantified has been the paramount goal, the definition of ECD is not yet uniform among centers.

In conclusion, this study suggests that long-term outcomes in recipients with high MELD scores (>30) can be improved by keeping the CIT below 6 h, as far as possible, and avoiding ABO-I LT. The use of ECD donors with factors such as old age, MVS, and other serological indicators may be allowed under close postoperative monitoring, which greatly improves the utilization and outcomes of ECD and reduces mortality on the waiting list.

## Data Availability Statement

The raw data supporting the conclusions of this article will be made available by the authors, without undue reservation.

## Author Contributions

MY, AK, DL, XW, CX, QW, and XX conceived and designed the experiments. MY, AK, CX, RW, BP, and BC collected the data. MY, AK, DL, XW, and ZZ analyzed the data. MY, AK, and DL wrote the paper. JC, SZ, and XX supervised the paper. All authors contributed to the article and approved the submitted version.

## Funding

This research was partially supported by National Natural Science Funds for Distinguished Young Scholar of China (No. 81625003); Key Program, National Natural Science Foundation of China (Nos. 81930016, 81570589, and 81702858).

## Conflict of Interest

The authors declare that the research was conducted in the absence of any commercial or financial relationships that could be construed as a potential conflict of interest.

## Publisher's Note

All claims expressed in this article are solely those of the authors and do not necessarily represent those of their affiliated organizations, or those of the publisher, the editors and the reviewers. Any product that may be evaluated in this article, or claim that may be made by its manufacturer, is not guaranteed or endorsed by the publisher.

## Abbreviations

ABO-I: ABO-incompatible
ALB: albumin
ALT: alanine aminotransferase
AST: aspartate aminotransferase
BMI: body mass index
Bun: blood urea nitrogen
C-index: concordance index
CIT: cold ischemia time
Cr: creatinine
DCD: donor after cardiac death
DDLT: deceased donor liver transplant
EAD: early allograft dysfunction
ECD: extended criteria donor
ESLD: end-stage liver disease
GRWR: graft-to-recipient weight ratio
GS: graft survival
GSR: graft survival rate
HBV: hepatitis B virus
HCC: hepatocellular carcinoma
HCV: hepatitis C virus
HL: Hosmer-Lemeshow
HR: hazard ratio
ICU: intensive care unit
INR: international normalized ratio
IQR: interquartile range
LT: liver transplantation
MELD: model for end-stage liver disease
MMF: mycophenolate mofetil
MVS: macrovesicular steatosis
POD: postoperative day
SCD: standard criteria donor
SD: standard deviation
TB: total bilirubin
WIT: warm ischemia time.
